# Fumonisin B1 Accumulates in Chicken Tissues over Time and This Accumulation Was Reduced by Feeding Algo-Clay

**DOI:** 10.3390/toxins13100701

**Published:** 2021-10-02

**Authors:** Julia Laurain, Didier Tardieu, Maria Matard-Mann, Maria Angeles Rodriguez, Philippe Guerre

**Affiliations:** 1 Olmix S.A., ZA du Haut du Bois, 56580 Bréhan, France; JLaurain@olmix.com (J.L.); mmatard@olmix.com (M.M.-M.); mrodriguez@olmix.com (M.A.R.); 2 National Veterinary School of Toulouse, ENVT, Université de Toulouse, 31076 Toulouse, France; didier.tardieu@envt.fr

**Keywords:** fumonisin, feed, food, muscle, liver, residues, algo clay, broiler chicken

## Abstract

The toxicokinetics of the food and feed contaminant Fumonisin B (FB) are characterized by low oral absorption and rapid plasma elimination. For these reasons, FB is not considered to accumulate in animals. However, recent studies in chicken and turkey showed that, in these species, the hepatic half-elimination time of fumonisin B1 (FB1) was several days, suggesting that FB1 may accumulate in the body. For the present study, 21-day-old chickens received a non-toxic dose of around 20 mg FB1 + FB2/kg of feed to investigate whether FB can accumulate in the body over time. Measurements taken after four and nine days of exposure revealed increased concentrations of sphinganine (Sa) and sphingosine (So) over time in the liver, but no sign of toxicity and no effect on performances were observed at this level of FB in feed. Measurements of FB in tissues showed that FB1 accumulated in chicken livers from four to nine days, with concentrations of 20.3 and 32.1 ng FB1/g observed, respectively, at these two exposure periods. Fumonisin B2 (FB2) also accumulated in the liver, from 0.79 ng/g at four days to 1.38 ng/g at nine days. Although the concentrations of FB found in the muscles was very low, an accumulation of FB1 over time was observed in this tissue, with concentrations of 0.036 and 0.072 ng FB1/g being measured after four and nine days of exposure, respectively. Feeding algo-clay to the chickens reduced the accumulation of FB1 in the liver and muscle by , approximately 40 and 50% on day nine, respectively. By contrast, only a weak non-significant effect was observed on day four. The decrease in the concentration of FB observed in tissues of chickens fed FB plus algo-clay on day nine was accompanied by a decrease in Sa and So contents in the liver compared to the levels of Sa and So measured in chickens fed FB alone. FB1 in the liver and Sa or So contents were correlated in liver tissue, confirming that both FB1 and Sa are suitable biomarkers of FB exposure in chickens. Further studies are necessary to determine whether FB can accumulate at higher levels in chicken tissues with an increase in the time of exposure and in the age of the animals.

## 1. Introduction

Fumonisins are mycotoxins produced by *Fusarium* that are found in food and feed all over the world [1,2,3]. Among fumonisins, fumonisin B (FB) is the most widespread and the most studied, and among FB, fumonisin B1 (FB1) and to a lesser extent fumonisin B2 (FB2) are the most abundant and the most toxic [4]. The toxicity of FB in mammals varies strongly with the animal species in terms of dose, length of exposure and clinical signs [3,5,6]. Leucoencephalomacia occurs in horses, who are one of the most sensitive species to FB, whereas pulmonary edema has been reported in pigs, and liver damage has been found in all animal species, including avian species, which are considered to be relatively resistant to FB. In rodents, FB1 is a known carcinogen that induces renal tubule and hepatic tumors in male rats and female mice, respectively [7]. In humans, consumption of FB has been associated with increased incidence of esophageal cancer and the International Agency for Research on Cancer has classified FB (group 2B) as “possibly carcinogenic to humans” [8]. Consequently, a tolerable daily intake for human consumption of FB has been established, and maximum tolerable levels of FB have been fixed in feed and in raw materials intended for animal consumption [3,9]. The recommendation on the presence of FB in complete feeding stuffs for poultry was 20 mg FB1 + FB2/kg.

One of the paradoxes related to the toxicity of FB in animals concerns the toxicokinetics of FB at the onset of mycotoxicosis. Most studies on animals concluded that FB toxicity is cumulative [3,5]. In avian species, prolonged exposure of ducks and turkeys to low doses of FB resulted in a gradual increase in sphinganine (Sa) and sphingosine (So) bases over time in the liver, Sa and So being recognized markers of FB exposure and toxicity [10,11]. However, toxicokinetic studies conducted both in avian species and in mammals revealed that FB is rapidly eliminated from the blood, and persistence of FB in animals was considered to be negligible [3,12]. The apparent paradox between the cumulative toxicity of FB and their rapid plasma elimination may in fact be related to the lack of sensitivity of the analytical methods used. Indeed, a recent study conducted using immuno-affinity cleanup of tissue samples followed by UHPLC-MSMS detection revealed that FB fed at low levels persisted in the liver of chickens and turkeys several days after exposure to the contaminated diet was stopped [13]. The half-life of elimination of FB1 from the liver calculated in this study was 66 h in chicken, suggesting that FB can accumulate in this tissue. 

Because FB are toxic compounds, several strategies have been developed to reduce their concentration in feed [14]. In 2009, the European Union (EU) approved the use of a new group of feed additives defined as ‘substances that can suppress or reduce the absorption, promote the excretion of mycotoxins or modify their mode of action’ [15]. Among them, adsorbing agents are one of the most often studied, clays and their derivatives being a key group routinely used in feed, especially to prevent the absorption of aflatoxins [15]. Moreover, clays, smectites, and their derivatives have also been seen to reduce FB toxicity and to decrease urinary biomarkers of FB [16,17,18,19,20,21]. Clays are phyllosilicates whose physico-chemical properties and adsorption capacities can vary according to their mining source and the spacing between the layers. Increasing the interlayer spacing generally increases the adsorption capacity of clays and modified clays tend to have higher mycotoxin-sequestering capacity [22,23]. Algo-clay is a modified clay adsorbent developed in a patented process (Olmix S.A., Brehan, France) using water-soluble polysaccharides extracted from marine green algae [24] and montmorillonite (layered clay). These water-soluble polysaccharides act as pillars between clay layers and increase the inter-laminar space up to 3 nm. Algo-clay has been reported to adsorb mycotoxin and reduce toxicity [25]. However, the efficiency of algo-clay in chicken fed FB at the maximum tolerated level defined by the EU guideline for avian feed has not yet been investigated. 

The first objective of this study was thus to assess whether FB can accumulate in tissue over time in chickens with a dose of FB in feed near the maximum level defined by the EU guidelines. The second objective was to investigate the consequence of feeding algo-clay with FB concentrations in tissue and on Sa and So levels in the liver.

## 2. Results and Discussion

### 2.1. Experimental Diets and Study Design

Different corn-soybean diets were prepared to best meet the nutritional needs of the chickens as described in Table S1. Diets containing algo-clay alone were used to control effects on performance because previous studies reported that detoxifying agents can reduce feed intake and growth [26]. The concentrations of fumonisin in the different diets are listed in Table 1. Only traces of FB were found in the control diet and in the diet containing algo-clay alone, whereas concentrations of FB1 + FB2 in the diet containing FB and in the diet containing FB + AC were 20.7 and 19.5 mg/kg, respectively. All other mycotoxins measured were at the trace level or below the detection limit (Table S2). After a growth period of 12 days, 70 chicken were divided into seven groups of 10 chickens. Each group was then divided into two pens each containing five chickens. Control diet and diets containing algo-clay, FB, and FB plus algo-clay were fed to the chickens for four to nine days until the chickens reached 20 or 21 days of age, as explained in Table S3. Slaughtering was performed on two consecutive days to minimize the difference in the time between the last meal and death that could influence the amount of FB1 in tissues [27]. Chickens exposed to algo-clay, FB and FB plus algo-clay for four days and one pen of five chickens fed the control diet were slaughtered when they reached 21 days of age. Chickens exposed for nine days and the other pen of five chickens fed the control diet were slaughtered when they were 22 days old (Table S3). 

### 2.2. Performances and Organ Weight

Neither mortality nor signs of mycotoxicosis were observed in this study. This result is in agreement with the results of several studies in chickens fed FB in feed at a level of near 20 mg FB1 + FB2/kg and, according to EU guidelines, concerning the maximum tolerated level of FB in feed [3,28,29]. As shown in Table 2, no significant difference in body weight (BW), feed intake (FI) and feed conversion ratio (FCR) were observed among groups. Post-mortem examination failed to reveal any macroscopic alteration in any of the chickens, and no difference in the weight of the liver, gizzard and heart was observed among groups (Table 2). The lack of an effect of FB on performance and organ weight in chickens at a dose near 20 mg FB1 + FB2/kg feed is in agreement with data in the literature, while the lack of an effect on the performance of algo-clay fed alone confirmed no unspecific effects of this adsorbing agent as reported in previous studies in pigs [3,28,29,30]. 

### 2.3. Plasma Biochemistry and Sphingoid Bases

Several variables used as markers of liver and kidney function were measured in plasma (Table 3). No significant differences in uric acid, cholesterol, proteins, albumin, or globulin concentrations were observed among groups. The activities of alanine aminotransferase (ALT), aspartate aminotransferase (AST), lactate dehydrogenase (LDH), creatinine phosphokinase (CPK), and alkaline phosphatases (PAL) in plasma remained unchanged regardless of diet. These results confirm those obtained in several studies in chicken in which feeding FB at a concentration in feed close to 20 mg FB1 + FB2/kg revealed no alteration in plasma biochemistry [3,28,29]. 

Sphinganine (Sa) and sphingosine (So) were measured in the livers to reveal the effects of FB on sphingolipid metabolism, and the Sa:So ratio was calculated. No statistical difference in Sa and So contents in the liver was observed among the groups not exposed to FB except for a decrease in So in chickens fed algo clay for four days (and which was no longer observed after nine days of exposure). This decrease was accompanied by a numerically non-significant decrease in Sa, leading to no significant difference in Sa/So among groups not exposed to FB. Feeding FB for nine days led to a significant increase in Sa and So in liver compared to controls not exposed to the toxin, but the increase was significantly lower in chickens fed FB plus algo-clay than in chickens fed FB alone. The amounts of Sa after four days of exposure were between those measured in controls not exposed to the toxin and in chickens fed FB for nine days. At four days of exposure, no significant difference of Sa/So was observed between the group fed FB plus algo-clay and the group fed FB alone. The effects of FB on Sa, So and Sa/So are in agreement with most data in the literature on broilers fed this level of FB in feed [29,31]. It should be noted that in the present study, both Sa and So increased in agreement with previous data obtained not only in chickens but also in ducks and turkeys [10,11,32]. An increase in both Sa and So is actually accepted to be a consequence of inhibition of ceramide synthases due to FB: levels of So generally increase later and less than Sa [6]. The results obtained in the present study are in agreement, as a 376% increase in Sa was observed at nine days of exposure while the increase in So over the same period was 164%. The effect of algo-clay on Sa and So we observed is also in agreement with data obtained with calcium montmorillonite clay, previously reported to decrease biomarkers of FB1 toxicity in cell culture and in vivo [16,17].

### 2.4. FB in Liver of Chickens Fed FB Alone

**Figure 1:**
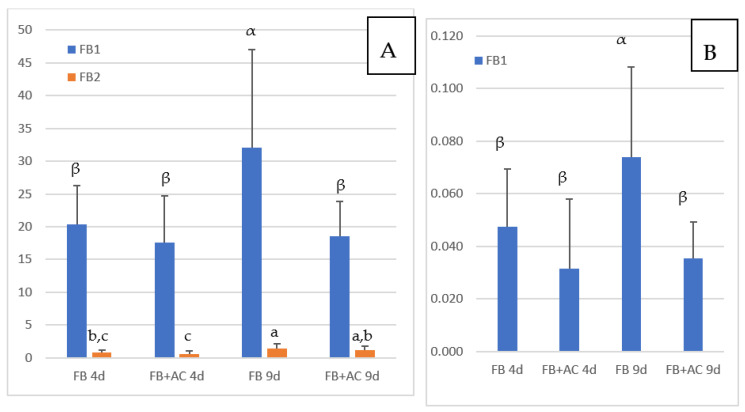
Concentrations of FB measured in chickens fed 4 and 9 days (d) with diets containing fumonisins B (FB) and fumonisins B + algo-clay (FB + AC). (**A**): liver; (**B**): Muscle. Results are expressed as Means ± SD of 10 determinations. ANOVA revealed significant difference among groups (*p* < 0.05). Different letters in the same row indicate statistically different groups (Duncan, *p* < 0.05).

The concentrations of FB1 and FB2 in the livers of chickens fed FB are reported in Figure 1A. FB3 was generally below the LOQ and is not shown in the figure. The concentrations of FB1, FB2, and FB3 in chickens not exposed to FB were below the LOD except in three animals, in which non-quantifiable levels of FB1 were detected. As shown in Figure 1, a mean FB1 concentration of 20.3 ng/g liver was observed in chickens fed FB for four days, and the concentration increased to 32.1 ng/g at nine days of exposure. The difference between the two groups was significant. Although several studies already reported FB1 in the livers of chickens fed FB, this is the first study to show that FB1 can concentrate in the liver with an increase in exposure times. The concentration of 32.1 ng FB1/g liver obtained after nine days of exposure was lower than the concentration of 44.7 ng/g reported in chickens fed 21 mg FB1/kg diet over a period of 35 days [33]. The concentrations of FB1 in liver measured in this study are also in agreement with those reported in different studies conducted in chickens and turkeys with different concentrations of FB1 in feed over a period of 14 days, and in an older study on turkey where a concentration of 117 ng FB1/g of liver was reported after feeding 20 mg FB1 + FB2/kg for a period of 63 days [13,33,34]. The concentration of 32.1 ng FB1/g measured in this study after nine days of exposure to a diet containing 15.2 mg FB1/kg is similar to the concentration of 30.3 ng/g liver reported in chickens fed 10.5 mg FB1/kg for 56 days [27]. 

**Figure 2:**
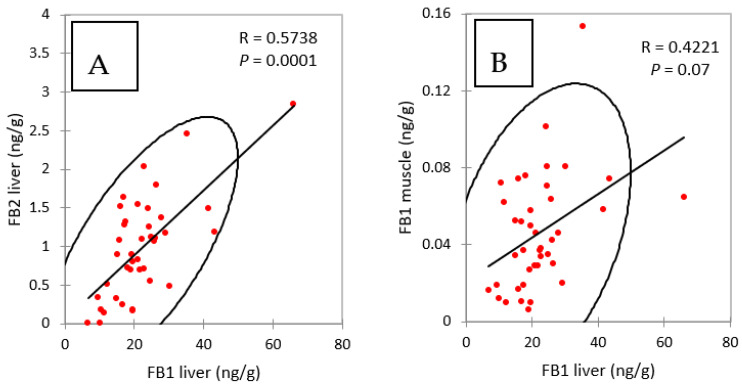
Spearman correlations between (**A**) FB1 and FB2 in liver and (**B**) FB1 in liver and FB1 in muscle, measured in chickens fed for 4 and 9 days with diets containing fumonisins B and fumonisins B + algo-clay. R = coefficient of correlation, *P* = *p* value.

The concentrations of FB2 in the livers of chickens fed FB over a period of four and nine days were notably lower than the concentrations of FB1 (Figure 1A). However, like FB1, FB2 appeared to accumulate in this tissue over time, as it increased from 0.79 ng/g at four days of exposure to 1.38 ng/g at nine days of exposure. As shown in Figure 2A, a significant correlation was observed between FB1 and FB2 in livers (Spearman, *p* = 0.0001). Although the ratio of FB1 to FB2 was lower than previously reported, these results are consistent with the lower concentrations of FB2 in feed than of FB1 (Table 1), and also in agreement with previous studies that reported a low level of FB2 in liver [13,27,33]. 

The evidence for FB accumulation in the liver differs strongly from its rapid plasma elimination. In the poultry species, the half-time of elimination of FB1 from the blood varies from 70 to 214 min in broilers, laying hens, ducks and turkeys while half-times of elimination of 12 and 32 min for FB2 have been reported in turkeys and ducks [12,35]. The rapid elimination of FB from the blood in avian species is in agreement with all the data in the literature on the toxicokinetics of FB [3]. However, some works conducted on liver suggested that the elimination of FB1 from this organ may take longer than from blood. A study in pigs fed a diet containing 45 mg FB1/kg for 10 days revealed that FB1 was still detectable in the liver 10 days after exposure to the toxin was stopped [36]. More recently, a study on broilers and turkeys fed a low dose (6 mg FB1/kg feed) revealed half-times of elimination of FB1 in the liver of 66 and 124 h, respectively [13]. 

The interest of our result showing that FB1 can accumulate in the liver goes beyond the sole knowledge of FB1 toxicokinetics in this tissue. Indeed, rapid elimination of FB1 from the body was not consistent with the results of most of the studies conducted on the sub-acute toxicity of FB, which revealed that toxicity of FB was cumulative. The first evidence for cumulative toxicity of FB1 was obtained in horses in which most cases of equine leucoencephalomalacia were observed after 14–21 days of exposure, but the onset of disease may occur as early as seven days after administration of FB or as late 90 days or more [4,37,38]. Cumulative toxicity of FB was then reported in different animal species, including avian ones [12]. Another important consequence of the bioaccumulation of FB1 in liver concerns human exposure. Indeed, it is generally accepted that human exposure to FB via food of animal origin is low compared to human exposure through plants, particularly corn and its byproducts [3,9,31]. The demonstration of the cumulative properties of FB1 in the liver of broilers suggests that the same could occur in other edible tissues such as muscle, and should be taken into account when evaluating human exposure to FB. 

### 2.5. FB in Muscle of Chickens Fed FB Alone

Concentrations of FB1 measured in muscle are reported in Figure 1B. Because the concentrations of FB1 measured in this study were lower than the LOQ of 0.25 ng/g previously defined for FB1 in muscle [33], we conducted a validation study of the recovery of FB in blank muscles spiked at two concentrations of 0.036 and 0.072 ng FB/g. At these concentrations, FB2 and FB3 were not quantifiable, while mean recovery of FB1 was 78 and 97%, respectively. Relative standard deviation (RSD) measured at 0.036 and 0.072 ng FB1/g muscle was 23% and 20% respectively. This complementary validation study indicated that the LOQ of FB1, defined at the lowest concentration validated, can be extended to 0.036 ng/g muscle, while the LOQ of FB2 and FB3 remained unchanged at 0.25 ng/g [33]. 

Figure 1B shows that concentrations of FB1 in muscle increased over time from 0.047 at four days of exposure to 0.074 ng/g at nine days. The difference between these two groups was significant, and the increase in the concentration in muscle over time was consistent with what was observed in the liver. Also, as shown in Figure 2B, a weak but significant correlation was observed between FB1 in liver and FB1 in muscle in this study (Spearman, *p* = 0.07). Only a few studies have reported the presence of FB1 in chicken muscle. In one study a concentration of 17.5 ng FB1/g was measured after FB1 was fed at a concentration of 21 mg/kg feed over a period of 35 days [33]. In another study, 2 ng FB1/g muscle were measured after feeding 10.5 mg FB1/kg over a period of 56 days [27]. Taken together, these results suggest that concentration of FB1 in muscle is highly variable in comparison to that reported in the liver. Together, the level of FB in the feed, the length of exposure and probably the age of the animals may influence the persistence of FB1 in muscle: the few data available today suggest that FB1 can accumulate in muscle over time as it does in the liver.

### 2.6. FB in Tissues of Chickens Fed FB Plus Algo-Clay

Figure 1A shows the concentrations of FB1 and FB2 in the liver of chickens fed FB plus algo-clay measured after four and nine days of exposure. After four days of exposure, only a slight difference was observed in the mean concentration of FB1 in the livers of chickens fed FB plus algo-clay compared to chickens fed FB alone. By contrast, after nine days of exposure, the concentration of FB1 in chickens fed FB plus algo-clay was 18.6 ng/g liver versus 32.1 ng/g in chickens fed FB alone. The difference between the two groups was significant, and feeding algo-clay enabled a 42% decrease in the concentration of FB1 in the liver compared to the concentration measured in chickens fed FB alone. Concerning muscle, a numeric decrease in the concentration of FB1 was observed after four days of exposure in chickens fed FB plus algo-clay compared to chickens fed FB alone, but the difference was not significant. By contrast, at nine days of exposure, the concentrations of FB1 were 0.035 ng/g in chickens fed FB plus algo clay and 0.074 ng/g in chickens fed FB alone, and the difference between the two groups was significant. Compared to chickens fed FB alone, feeding algo-clay enabled a 53% decrease in the concentration of FB1 in muscle.

**Figure 3:**
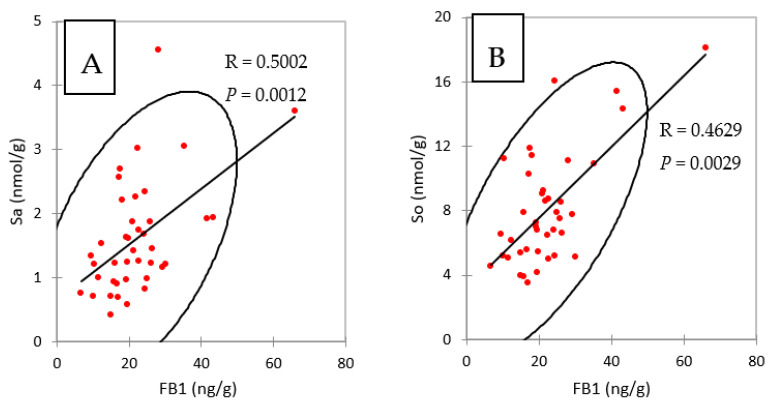
Spearman correlations between (**A**) FB1 and sphinganine (Sa) and (**B**) FB1 and sphingosine (So), measured in liver of chickens fed 4 and 9 days with diets containing fumonisins B and fumonisins B + algo-clay.

Taken together, the results obtained in liver and in muscle showed that concentrations of FB1 in chickens fed FB plus algo-clay for nine days were close to the concentrations of FB1 found in chickens fed FB alone for four days, suggesting that feeding the algo-clay prevented the accumulation of FB1 in these tissues. No previous data on algo-clay and FB are available to enable us to compare this result with others. However, clays and their derivates have been reported to reduce FB toxicity in cell cultures and in vivo in animals [18,19,20,21]. Calcium montmorillonite clay was also reported to reduce biomarkers of fumonisins [16,17]. The latter results are consistent with the observation that the concentrations of FB1 in the livers of chickens fed FB and of chickens fed FB plus algo-clay paralleled the concentrations of Sa and So in liver (Table 3 and Figure 1). As shown in Figure 3, a significant correlation was observed in the present study between FB1 and Sa and So in livers, confirming that FB1 and Sa are suitable biomarkers to reveal exposure to FB.

In conclusion, this study demonstrated for the first time that fumonisins can accumulate in the liver and muscle of 21-day-old chickens with no sign of toxicity or decrease in performance when fed for four and nine days at a concentration near the maximum level defined by EU guidelines. This study also demonstrated for the first time that feeding algo-clay with fumonisins reduced the accumulation of FB1 by around 40% in liver and by 50% in muscle. Further studies are necessary to determine whether FB still accumulates in tissues over longer periods of exposure and whether the age of animals could influence the amount of FB that accumulates in muscles. 

## 3. Material and Methods

### 3.1. Chemicals and Reagents

All reagents were purchased from Sharlab (Sharlab S.L., Sentmenat, Spain), Fluka (Fluka, Buchs, Switzerland) and Sigma (Sigma Chemical Co, Saint Quentin Fallavier, France). Pure water, methanol, formic acid, and acetic acid were of LC-MS grade; all other reagents were of HPLC analytical grade. Standard solutions of FB1, FB2, FB3, [^13^C_34_]-FB1, [^13^C_34_]-FB2 and [^13^C_34_]-FB3 with certified concentrations of each analyte and all other mycotoxins dosed in feed and reported in Table S2 were purchased from Biopure (Romer Labs, 3131 Getzersdorf, Austria) and Sigma (Sigma Chemical Co, Saint Quentin Fallavier, France). Immunoaffinity FUMONIPREP columns were purchased from R-Biopharm (R-Biopharm Rhone LTD, Glasgow, Scotland). Sphinganine, sphingosine, and C20 sphinganine were purchased from Bertin (Bertin Technologies, Montigny le Bretonneux, France). Algo clay (Batch number R174P1E22) was provided by Olmix (Olmix S.A, 56580 Bréhan, France).

### 3.2. Experimental Diets and Analysis of Mycotoxins in Feed

Diets were formulated on a corn-soybean basis by Tecaliman (Tecaliman, 44323 Nantes, France) to best meet the nutritional needs of the chickens used in this study (Table S1). Diets containing FB were made of corn containing FB at an expected final concentration of 20 mg FB1 + FB2/kg. Algo clay was incorporated in the experimental diets at a concentration of 450 mg/kg. Mycotoxins in raw materials and in final diets were analyzed according to the AFNOR V03-110 recommendation [29,39]. The FB concentration in the experimental diets is reported in Table 1, while the other mycotoxins measured are reported in Table S2.

### 3.3. Animal Husbandry and Sample Collection

The study was carried out at Cebiphar (Cebiphar, 37230 Fondettes, France) as a randomized, parallel, mono-centric study attributed the number V9152. This study was conducted under project no. 2017062111426641 accepted by French Ministry of Higher Education, Research and Innovation on 6 November 2017. Ninety-two one-day-old chicks vaccinated against Gumboro and Marek diseases and coccidiosis were supplied by Boyé Accouvage (Boyé Accouvage, 79310 La Boissière en Gâtine, France). On day one, 84 healthy chickens in good physical condition were randomly housed in groups of six in 14 floor pens in one room (indoor housing) at the Cebiphar experimental facilities. The allocation was checked on day 10 to ensure homogeneity across pens and treatment groups. Seventy chicks were sorted by increasing body weights and then attributed a random number in blocks of seven. All the pens were designed to provide the chicks with similar environmental conditions (the same airspace). The bedding was wood shavings placed on a concrete floor. The animals were clinically observed at least daily from arrival to euthanasia.

Drinking water and feed were provided ad libitum in one feeder per pen throughout the experiment. On the first two days, the chicks were fed a starter feed. On day three, the control feed was progressively mixed with the starter feed to become the unique source of feed for all animals on day six to at least day 12. From day 13 or day 17 to the end of the study, feed containing FB, algo clay and FB plus algo clay were available ad libitum to the chickens according to the treatment group defined in Table S3. Feed intake was measured per pen from day 6 to day 12, day 13 to day 16, and day 17 to day 20 or 21. The chickens were weighed individually on days 1, 10, 17, and 21.

On day 21 or day 22, feed was removed eight hours before blood collection and euthanasia. Blood was sampled into EDTA tubes using single-use 22G sterile needles. The collected blood was centrifuged at 2,500 rpm for 10 min at +5 °C and the plasma collected in three 1-mL pre-labelled tubes. Euthanasia was carried out after blood collection by electrical stunning followed by bloodletting in compliance with European Directive EC 2010/63. All the animals were macroscopically examined for gross pathology. The liver, gizzard, heart, and breast muscle were collected and were placed in pre-labelled polypropylene vials. All samples were stored at −20 °C until analysis.

### 3.4. Experimental Blinding 

Feed distribution was not blinded at the test facility. Pens were identified with a letter for the treatment group and a number (two pens per treatment). All the biological samples were identified with the study number (V9152), the pen number, and the leg ring numbers of the chicken. All the samples were blind analyzed. The person who analyzed the samples was aware of how the chicken were grouped but not of which different feed was allocated to each pen. The blind ended after all the samples were analyzed. 

### 3.5. Biochemistry and Sphingosine and Sphinganine in Liver

The concentrations of uric acid, cholesterol, proteins, albumin, and globulins were measured with a clinical chemistry analyzer KONELAB 20 (Fisher Scientific SAS, 67400 Illkirch, France) according to the manufacturer’s instructions. The activity of lactate dehydrogenase (LDH, EC 1.1.1.27), alkaline phosphatase (ALP, EC 3.1.3.1), alanine aminotransferase (ALT, EC 2.6.1.2), aspartate aminotransferase (AST, EC 2.6.1.1), and creatinine phosphokinase (CPK, EC 2.7.3.2) was measured with the same apparatus and is expressed in UI/L of plasma.

Fractions of liver used for the analysis of the sphingoïd bases were prepared at 4 °C by homogenization with an Ultra Turrax of 1 g of liver in 3 mL of phosphate buffer (0.1 M, pH 7.4) containing Tris acetate, potassium chloride, EDTA and butylated hydroxytoluene. After centrifugation at 3000× *g* for 15 min the supernatant was collected and stored at −80 °C until analysis. Free sphinganine (Sa) and sphingosine (So) were measured by HPLC after derivatization with orthophtaldialdehyde (OPA) as previously described by Riley et al. with slight modifications [40,41]. Briefly, 0.5 nmol of C20 sphinganine used as internal standard (IS) were added to 100 μL of liver homogenate, the lipids were then hydrolyzed for 1 h at 37 °C by alkaline methanolic-chloroform to liberate the sphingoïd bases. The chloroform phase was washed twice with alkaline water before being evaporated to dryness. The dried extracts were then suspended in ethanol and placed in an automate (ICS M2200 solvent delivery module) for derivatization with OPA before injection into the HPLC system (ICS, Toulouse, France). The sphingoïd bases were detected using a programmable fluorescence detector (RF10 AXL Shimazu, Kyoto, Japan) after separation of the analytes on a Prontosil C18 column (250 × 4.6 mm) and a C18 precolumn (Bischoff, Leonberg, Germany). The chromatographic conditions were as follows: liquid phase: methanol-water (90:10), flow rate: 1.25 mL/min, excitation wavelength: 335 nm, emission wavelength: 440 nm. The concentrations of the analytes were calculated by linear regression from standard solutions injected daily. Concentrations of Sa and So were corrected by the recovery rate measured for the IS. 

### 3.6. Fumonisins in Tissues

The concentrations of FB1, FB2 and FB3 in tissues were determined by UHPLC-MS/MS after immunoaffinity clean-up of samples using [^13^C_34_]-FB1, [^13^C_34_]-FB2 and [^13^C_34_]-FB3 as IS as previously described [33]. Briefly, 25 mg of NaCl, 4 mL of water/acetonitrile/methanol (2:1:1) and 12.5 ng of IS were added to 1 g of liver before homogenization with an Ultra Turrax. Muscle was prepared in the same way except 5 g of tissue were homogenized in 20 mL of water/acetonitrile/methanol (2:1:1). Homogenized samples were stirred for 2 h on a stir table, centrifuged, and the supernatant was washed with 8 mL of hexane. After centrifugation the organic phase was removed. An aliquot of the aqueous phase was diluted in 1 mM pH 7.3 PBS and passed through a FUMONIPREP column according to the manufacturer’s instructions. Extracts were stored as dry residue at −20 °C until analysis. 

The analytes were separated on an Agilent 1260 UHPLC (Agilent, Santa Clara, CA, USA) on a Poroshell 120 column (3.0 × 50 mm, 2.7 μ) using a gradient of elution and a mobile phase composed of methanol and water, each containing 0.1% formic acid (*v*/*v*) [33]. Detection was conducted after positive electrospray ionization at 300 °C with a MS/MS 6410 triple quadrupole detector from Agilent with the following flow conditions: gas delivery rate 10 L/min, nebulization at 25 psi and capillary voltage at 4000 V. Agilent MassHunter quantitative analysis software was used to analyze the chromatograms. For each analyte, the most abundant product ion was used as quantifier. Two transitions were used as qualifiers for FB1, FB2, and FB3 whereas one qualifier was used for the IS. The method was linear (Fisher’s test, *p* < 0.01, and r2 ≥ 0.99) with good accuracy (RSR = relative standard deviation of 20%) from 2 to 100 ng/mL. Good recovery of FB (78 to 126%, RSD < 20%) was observed in liver over a concentration range of 0.25 to 100 ng/g for FB1 and 0.25 to 25 ng/g for FB2 and FB3. Good recovery (83 to 121%, RSD < 20%) was also observed for FB1 in muscle: over 0.25 to 25 ng/g and for FB2 and FB3 over 0.25 to 5 ng/g [33]. The LOQ, defined as the lowest validated concentration, was 0.25 ng/g for FB1, FB2 and FB3 in liver and in muscle. The variation of the qualifier ratio in the samples had to be less than 20% compared to the qualifier ratio measured in the standards. The retention time in the samples had to vary less than 5% from the retention time measured in the standards. 

Because the concentrations of FB1 measured in muscle in this study were <0.25 ng FB1/g, a complementary validation study was conducted. Briefly, the linearity of the method of analysis was first measured on standard solutions of FB at 0.09, 0.039, 1.56, and 6.25 ng/mL (*n* = 5). Despite the very low concentration assayed, good linearity (r^2^ = 0.9901, *p* < 0.0001) was observed for FB1 (Figure S1). The recovery of FB in blank samples obtained from chickens fed the control mycotoxin-free diet was measured in 5 g of muscle spiked prior to the extraction at 0.036 and 0.072 ng FB/g and 2.5 ng IS/g, as previously described. Due to the very low concentrations assayed, results in muscles were considered acceptable when variation of the qualifier ratio was less than 25% of the ratio measured in the standards. 

### 3.7. Statistical Analysis

Data are reported as means ± SD. After checking the homogeneity of variance (Hartley’s test), one-way ANOVA was performed to compare groups. When significant differences were observed among groups (*p* < 0.05), means were compared using Duncan’s multiple range test. Different letters in the same row identify statistically different groups (*p* < 0.05). The linearity of the analytical methods was checked using a Fisher’s test. Correlations between variables were measured with a Spearman’s test. All statistical analyses were performed using XLSTAT Biomed (Addinsoft, 33000 Bordeaux, France).

## Figures and Tables

**Table 1:** Levels of fumonisins in the experimental diets ^1^.

Mycotoxin	Control	AC	FB	FB + AC
Fumonisin B1	0.04 ± 0.01	0.03 ± 0.01	15.2 ± 4.3	14.0 ± 3.3
Fumonisin B2	0.02 ± 0.01	<0.01	5.59 ± 0.56	5.57 ± 0.48
Fumonisin B3	<0.01	<0.01	0.89 ± 0.15	0.89 ± 0.14

^1^ Results are expressed as mean ± SD of 4 determinations. ANOVA revealed no significant difference between groups that contained FB (*p* > 0.05). FB = fumonisins B diet; AC = algo-clay diet; FB + AC = fumonisins B + algo-clay diet. Detailed composition of the experimental diets and contents of mycotoxins other than FB are reported in Tables S1 and S2, respectively.

**Table 2:** Performances and organ weights measured in chickens fed for four and nine days (d) with control diet and diets containing fumonisins (FB) and algo-clay (AC) and fumonisins B + algo-clay (FB + AC).

Variable	Control	AC 4d	AC 9d	FB 4d	FB + AC 4d	FB 9d	FB + AC 9d
BW D10	274 ± 26	278 ± 40	280 ± 28	272 ± 35	276 ± 38	274 ± 31	278 ± 27
BW D17	661 ± 59	703 ± 109	696 ± 67	738 ± 98	648 ± 115	673 ± 90	688 ± 81
BW D21	994 ± 117	985 ± 141	1050 ± 115	1091 ± 132	943 ± 180	1031 ± 140	1016 ± 114
FI D12	2407 ± 8	2562 ± 107	2509 ± 56	2611 ± 138	2556 ± 60	2582 ± 26	2627 ± 111
FI D13-D16	1615 ± 29	1744 ± 57	1772 ± 4	1794 ± 33	1717 ± 158	1702 ± 108	1798 ± 46
FI D17-D21	2435 ± 470	2000 ± 16	2574 ± 409	2472 ± 195	2219 ± 269	2563 ± 652	2536 ± 670
FCR	1.3	1.28	1.31	1.26	1.38	1.33	1.37
Liver (%)	2.09 ± 0.169	2.059 ± 0.203	2.045 ± 0.186	2.148 ± 0.236	2.22 ± 0.254	2.108 ± 0.325	2.321 ± 0.323
Gizzard (%)	2.384 ± 0.459	2.383 ± 0.203	2.328 ± 0.491	2.178 ± 0.384	2.57 ± 0.552	2.392 ± 0.446	2.631 ± 0.642
Heart (%)	0.569 ± 0.054	0.573 ± 0.059	0.577 ± 0.069	0.57 ± 0.061	0.564 ± 0.068	0.595 ± 0.119	0.608 ± 0.064

D: day of age; BW: body weight (g), mean ± SD, *n* = 10; FI: feed intake (g), mean ± SD for five animals, *n* = 2; FCR: feed conversion ratio calculated over the entire period. ANOVA revealed no significant difference among groups (*p* > 0.05).

**Table 3:** Plasma biochemistry and liver sphingoid bases measured in chickens fed for 4 and 9 days (d) with control diet and diets containing fumonisins (FB) and algo-clay (AC) and fumonisins B + algo-clay (FB + AC).

Variable ^1^	Control	AC 4d	AC 9d	FB 4d	FB + AC 4d	FB 9d	FB + AC 9d
Uric Acid ^2^	628 ± 263	590 ± 189	600 ± 173	393 ± 167	515 ± 267	551 ± 207	433 ± 149
Cholesterol ^3^	3.65 ± 0.62	3.5 ± 0.53	3.9 ± 0.58	3.85 ± 0.54	3.95 ± 1.79	3.81 ± 0.72	3.86 ± 0.34
Proteins ^4^	24.6 ± 1.9	25.2 ± 2.3	26.4 ± 2.1	26.7 ± 2.1	23.3 ± 6	27.8 ± 2.8	26.6 ± 2.2
Albumin ^4^	12.1 ± 0.8	12.6 ± 2.5	12.6 ± 0.8	12.6 ± 0.6	11.6 ± 2.1	13.3 ± 1.1	12.9 ± 0.9
Globulins ^4^	12.7 ± 1.3	12.6 ± 1.7	14.3 ± 2.7	14.2 ± 1.7	13 ± 1.2	14.4 ± 1.8	13.6 ± 1.8
AST ^5^	207 ± 26	252 ± 89	207 ± 32	226 ± 60	203 ± 77	225 ± 31	195 ± 32
ALT ^5^	8.2 ± 2.2	8.7 ± 1.95	9.38 ± 2.33	7.4 ± 1.58	10.7 ± 8.91	7.89 ± 2.15	7.3 ± 2.36
LDH ^5^	1512 ± 487	1865 ± 720	1448 ± 389	1609 ± 532	1453 ± 729	1388 ± 326	1317 ± 355
CPK ^5^	6169 ± 2629	5424 ± 2829	6363 ± 311	7554 ± 2424	3967 ± 3171	5866 ± 2728	6061 ± 2669
PAL ^5^	4527 ± 2285	5839 ± 1512	5128 ± 1809	5498 ± 2271	3586 ± 2031	4240 ± 1635	5778 ± 2979
Sa ^6^	0.66 ± 0.3 ^DE^	0.37 ± 0.18 ^E^	0.5 ± 0.18 ^DE^	0.93 ± 0.23 ^CD^	1.15 ± 0.45 ^C^	2.48 ± 0.96 ^A^	1.88 ± 0.71 ^B^
So ^6^	7.21 ± 3.08 ^BC^	3.74 ± 1.16 ^D^	8.11 ± 2.4 ^B^	6.63 ± 2.19 ^BC^	5.55 ± 1.51 ^CD^	11.86 ± 4.1 ^A^	8.14 ± 2.04 ^B^
Sa/So	0.1 ± 0.03 ^BC^	0.11 ± 0.06 ^BC^	0.07 ± 0.03 ^C^	0.15 ± 0.04 ^B^	0.21 ± 0.06 ^A^	0.24 ± 0.09 ^A^	0.23 ± 0.09 ^A^

^1^ Results are expressed as mean ± SD of 10 animals per group. ANOVA was performed to compare groups. When a significant difference was observed (*p* < 0.05), means were compared (Duncan). Different letters in the same row identify statistically different groups (*p* < 0.05); ^2^ in µmol/L; ^3^ in mmol/L; ^4^ in g/L; ^5^ in U/L: ^6^ in nmol/g. AST = aspartate aminotransferase; ALT = alanine aminotransferase; LDH = lactate dehydrogenase; CPK = creatinine phosphokinase; PAL = phosphatases alkaline; Sa = sphinganine; So = sphingosine.

## Data Availability

None of the data presented have been deposited in an official repository.

## Supplementary Materials

The following are available online at https://www.mdpi.com/article/10.3390/toxins13100701/s1, Table S1. Composition of feed and nutrient contents in the experimental diets. Table S2. Levels of mycotoxins other than fumonisins in the experimental diets. Table S3. Feed allocation to the different groups. Figure S1. Linearity of FB1 as solution of standard.

## Abbreviations

FB	Fumonisins B
FB1	Fumonisin B1
FB2	Fumonisin B2
FB3	Fumonisin B3
AC	AlgoClay
BW	Body Weight
FI	Feed Intake
FCR	Feed Conversion Ratio
Sa	Sphinganine
So	Sphingosine
OPA	orthophtaldialdehyde
IS	Internal Standard
IA	Immunoanity
RSD	Relative Standard Deviation
