# Supplementary material for: Fumonisin B1 Accumulates in Chicken Tissues over Time and This Accumulation Was Reduced by Feeding Algo-Clay
Source: Toxins (Basel). 2021 Oct 2;13(10):701. doi: 10.3390/toxins13100701 (PMC8537492; doi:10.3390/toxins13100701)
Supplement: Supplementary file 1 [file toxins-13-00701-s001.zip › toxins-1393914-SI.pdf]

Supplementary Materials: Fumonisin B1 Accumulates in Chicken Tissues over Time and this Accumulation Was Reduced by Feeding Algo-Clay

Julia Laurain, Didier Tardieu, Maria Matard-Mann, Maria Angeles Rodriguez and Philippe Guerre

Table S1. Composition of feed and nutrient contents in the experimental diets[1].

Variable	Control	AC	FB	FB+AC
Corn	26.9	26.9	20	20
Corn with fumonisins[2]	0	0	6.9	6.9
Wheat	29.9	29.9	29.9	29.9
Soybean meal	32.3	32.3	32.3	32.3
Soybean seed	2.5	2.5	2.5	2.5
Soybean oil	2	2	2	2
Sodium bicarbonate	0.13	0.13	0.13	0.13
Phosphate bicarbonate	1.78	1.78	1.78	1.78
Carbonate	0.61	0.61	0.61	0.61
Sodium chloride	0.25	0.25	0.25	0.25
DL-Methionine	1.91	1.91	1.91	1.91
Lysine HCL	0.74	0.74	0.74	0.74
L-threonine	0.58	0.58	0.58	0.58
VHT 789NE[3]	0.4	0.4	0.4	0.4
Intercalated algo-clay	0	0.045	0	0.045

[1]Expressed in % dry matter: FB = fumonisins B diet, AC = algo-clay diet; [2]Fumonisin contents were 186.5, 76.55, and 18.5 mg/kg of FB1, FB2, and FB3, respectively; [3]Poultry additive premix providing vitamins and trace elements.

Table S2. Levels of mycotoxins other than fumonisins in the experimental diets[1].

Mycotoxin	Control	AC	FB	FB+AC
Moniliformin	<0.1	<0.1	<0.1	<0.1
Zearalenone	0.013	0.013	0.018	0.01
Alpha-zearalenol	<0.01	<0.01	<0.01	<0.01
Beta-zearalenol	<0.01	<0.01	<0.01	<0.01
Alpha-zearalanol	<0.01	<0.01	<0.01	<0.01
Beta-zearalanol	<0.01	<0.01	<0.01	<0.01
Deoxynivalenol (DON)	0.13	0.12	0.12	0.12
DON-3-glucoside	0.013	0.008	0.01	0.01
Deepoxy-DON	<0.01	<0.01	<0.01	<0.01
15 Acetyl-DON	0.013	0.013	0.008	0.008
3 Acetyl DON	<0.01	<0.01	<0.01	<0.01
Fusarenon X	<0.01	<0.01	<0.01	<0.01
Nivalenol	<0.01	<0.01	<0.01	<0.01
Diacetoxyscipenol	<0.01	<0.01	<0.01	<0.01
15 monoacetoxyscirpenol	<0.01	<0.01	<0.01	<0.01
T2 toxin	<0.01	<0.01	<0.01	<0.01

HT2 toxin	<0.01	<0.01	0.01	0.01
T2 tetraol	<0.02	<0.02	<0.02	<0.02
T2 triol	<0.02	<0.02	<0.02	<0.02
Roridin A	<0.01	<0.01	<0.01	<0.01
Verrucarol	<0.01	<0.01	<0.01	<0.01
Verrucarin A	<0.01	<0.01	<0.01	<0.01
Tenuazonic acid	<0.01	<0.01	<0.01	<0.01
Aflatoxin B1	<0.001	<0.001	<0.001	<0.001
Aflatoxin B2	<0.001	<0.001	<0.001	<0.001
Aflatoxin G1	<0.001	<0.001	<0.001	<0.001
Aflatoxin G2	<0.001	<0.001	<0.001	<0.001
Ochratoxin A	<0.001	<0.001	<0.001	<0.001
Ochratoxin alpha	<0.005	<0.005	<0.005	<0.005
Ochratoxin B	<0.001	<0.001	<0.001	<0.001
Verruculogen	<0.02	<0.02	<0.02	<0.02
Cyclopiazonic acid	<0.05	<0.05	<0.05	<0.05
Citrinin	<0.05	<0.05	<0.05	<0.05
Patulin	<0.01	<0.01	<0.01	<0.01
Sterigmatocystin	<0.01	<0.01	<0.01	<0.01
Ergocornin	<0.01	<0.01	<0.01	<0.01
Ergocristin	0.01	0.015	0.02	0.013
Ergocryptin	0.01	<0.01	<0.01	<0.01
Ergometrin	<0.01	<0.01	<0.01	<0.01
Ergosin	0.013	0.013	0.018	0.015
Ergotamin	0.02	0.013	0.025	0.025

[1] Expressed in mg/kg as the mean of 4 determinations; FB = fumonisin B diet; AC = algo-clay diet; FB+AC = fumonisin B + algo-clay diet.

Table S3. Allocation of feed to the different groups.

Group	D1 to D5	D6 to D12	D13 to D16	D17 to D20	D21	D22
Control	Control	Control	Control	Control	Slaughter	
				Control	Control	Slaughter
AC 4 days	Control	Control	Control	AC	Slaughter	
AC 9 days	Control	Control	AC	AC	AC	Slaughter
FB 4 days	Control	Control	Control	FB	Slaughter	
FB 9 days	Control	Control	FB	FB	FB	Slaughter
FB+AC 4 days	Control	Control	Control	FB+AC	Slaughter	
FB+AC 9 days	Control	Control	FB+AC	FB+AC	FB+AC	Slaughter

n= 12 / group (10 + 2 spares; 6 per pen) from D1 to D10, then n = 10 per group (5 per pen) until euthanasia. FB = fumonisin B diet; AC = algo-clay diet; FB+AC = fumonisin B + algo-clay diet.

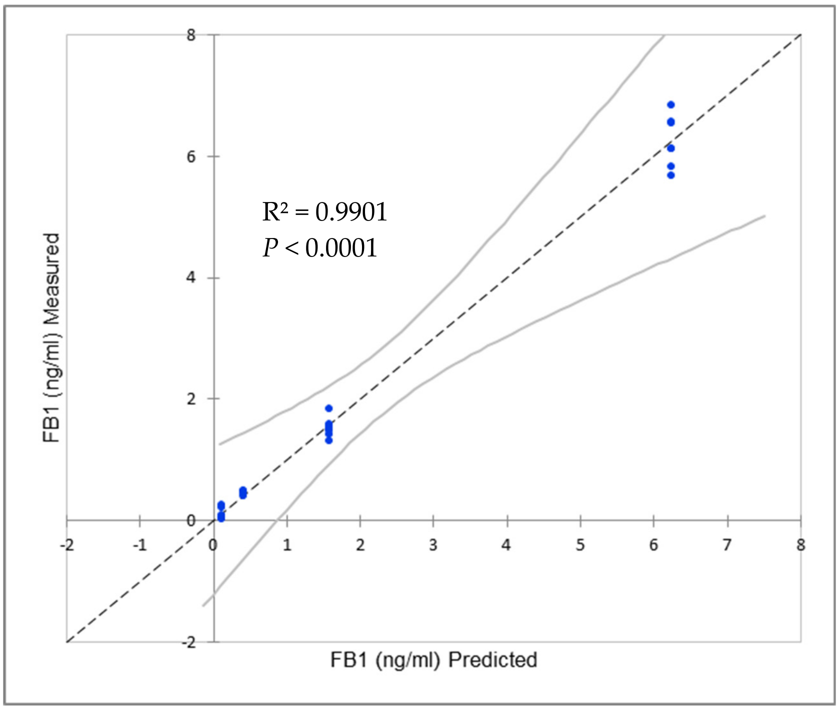

Figure S1. Linearity of FB1 as standard.
